# Exploring Multifactorial Relationships: Assessing the Correlation Between Cardiovascular Health Indicators and Metabolic Markers

**DOI:** 10.7759/cureus.59934

**Published:** 2024-05-08

**Authors:** Sandesh Shende, Jaishriram Rathored, Nisha Barole

**Affiliations:** 1 Clinical Research, Datta Meghe Institute of Higher Education and Research, Wardha, IND; 2 Central Research Laboratory and Molecular Diagnostics, Datta Meghe Institute of Higher Education and Research, Wardha, IND

**Keywords:** hypertension, diabetes mellitus, dyslipidemia, cardiovascular diseases, metabolic health

## Abstract

Background: The significant global health burden associated with cardiometabolic diseases necessitates a better understanding of the complex interrelationships between metabolic markers and cardiovascular health indicators. Crucial indicators of cardiovascular and metabolic health include metabolic markers such as uric acid and gamma-glutamyl transferase (GGT), as well as blood pressure (BP), triglycerides, total cholesterol, high-density lipoprotein (HDL), and fasting blood glucose.

Objective: This study aims to investigate the multifactorial relationships among several parameters such as age, BP, lipid profile, body mass index (BMI), fasting blood glucose levels, and specific metabolic enzymes.

Methods: A willing participant who visited the outpatient clinic and was in good health but did not have a history of cardiovascular disease was enrolled in the study. The collected data were subjected to a cross-sectional analysis. Various biochemical and health parameters such as height, weight, BMI, uric acid, triglycerides, HDL, BP, gender, age, and fasting blood glucose were analyzed, and a Pearson correlation coefficient analysis was performed to evaluate the correlations between these variables.

Results: Among the 50 study participants, significant Pearson correlations were observed between metabolic health markers such as BP (systolic and diastolic), fasting blood sugar, total cholesterol, triglycerides, HDL, and BMI. Additionally, a positive correlation was found between these metabolic parameters, including the levels of uric acid and liver enzyme GGT.

Conclusion: This study illustrates the intricate relationships among lipid profiles, liver enzymes, BP, and other metabolic health markers in the general population. Understanding these associations can help create focused interventions and individualized care plans for metabolic and cardiovascular disorders. Our findings address the complexity of cardiometabolic health and its management by identifying multifactorial risk factors linked to metabolic disorders and cardiovascular diseases.

## Introduction

Globally, cardiovascular diseases (CVDs) are the primary cause of morbidity and mortality. The development and progression of CVDs are influenced by metabolic health markers, including blood pressure (BP) and lipid profiles. Understanding the complex interplay between these metabolic markers is essential for effective prevention and management. This framework’s evaluation of the relationship between BP, lipid profiles, and additional metabolic markers in adult outpatients offers important insights into the mechanisms underlying CVDs in practical clinical settings. Metabolic disorders and CVDs are the world’s leading causes of morbidity and death, presenting enormous challenges to public health systems around the world [1]. The prevalence of these conditions is increasing despite advances in medical science and technology, which calls for a deeper understanding of their underlying mechanisms and interrelationships. Because cardiometabolic health is complex, multiple physiological, biochemical, and lifestyle-related factors interact in multiple ways [2]. CVDs are a group of illnesses that affect the heart and blood vessels. These illnesses include heart failure, peripheral arterial disease, coronary artery disease, and ischemic stroke [3]. According to the World Health Organization (WHO), CVDs are the primary cause of death worldwide and are responsible for approximately 17.9 million deaths per year [4]. Hypertension, dyslipidemia, diabetes mellitus, obesity, smoking, and a sedentary lifestyle are important risk factors for CVDs [5]. These risk factors usually coexist and operate in concert to promote the onset and progression of cardiovascular diseases. Furthermore, there is growing evidence that metabolic disorders like obesity, dyslipidemia, and insulin resistance are critical to the pathophysiology of CVDs, emphasizing the complex interactions between metabolic and cardiovascular pathways.

The dysregulation of glucose and lipid metabolism that characterizes metabolic disorders is a serious public health concern, with obesity and diabetes mellitus being the main contributors [6]. Hyperglycemia, the hallmark of diabetes mellitus, is a metabolic disorder that increases the risk of macrovascular complications such as peripheral vascular disease, coronary artery disease, and ischemic stroke [7]. Similarly, metabolic syndrome, a collection of disorders that includes abdominal obesity, insulin resistance, dyslipidemia, hypertension, and CVD, is significantly increased by obesity, which is defined as an abnormal or excessive buildup of body fat [8]. Effective risk assessment, prevention, and management require a thorough understanding of the multifactorial relationships between different physiological and biochemical parameters, given the complex etiology and pathophysiology of cardiometabolic diseases. Numerous traditional risk factors for CVDs, such as age, smoking, dyslipidemia, hypertension, and family history, have been identified in epidemiological studies. Nonetheless, new studies have shown the importance of investigating novel biomarkers and metabolic pathways related to metabolic homeostasis and cardiovascular health [9]. In recent years, the concept of precision or personalized medicine has gained popularity. Its goal is to customize medical interventions based on individual characteristics such as genetic composition, lifestyle choices, and biomarker profiles. Incorporating information from various sources, such as genetic, clinical, and environmental aspects, permits a comprehensive method for evaluating risk and managing illnesses.

BP is a vital indicator of cardiovascular health because it represents the force exerted by blood circulating through the walls of the arteries. Heart failure, stroke, and coronary artery disease are among the cardiovascular diseases for which elevated BP, especially hypertension, is a recognized risk factor [10]. On the other hand, normal BP is a sign of a sound cardiovascular system and proper metabolic equilibrium [11]. The measurement of blood glucose levels following an overnight fast, known as fasting blood sugar, is a crucial diagnostic criterion for both diabetes mellitus and impaired glucose tolerance. In addition to indicating poor glucose metabolism, dysregulated blood glucose levels also play a role in the emergence of insulin resistance, atherosclerosis, and other metabolic disorders [12]. Triglycerides, HDL, and total cholesterol are just a few lipid components included in the lipid profiles. The two essential lipid fractions linked to atherosclerosis and cardiovascular disorders are total cholesterol and triglycerides. An increased risk of coronary artery disease and other cardiovascular complications is linked to elevated levels of these lipids [13]. Conversely, HDL cholesterol, commonly known as "good cholesterol," serves a preventive function by aiding in the elimination of surplus cholesterol from the blood, consequently mitigating the likelihood of atherosclerosis and cardiovascular events. Liver enzymes, like uric acid and GGT, shed light on the body’s metabolic processes and liver function. GGT is an enzyme that is mostly located in the liver and is involved in the metabolism of other peptides and glutathione. Increased GGT levels are a sign of oxidative stress and liver dysfunction and are linked to several metabolic diseases, alcoholism, and liver injury [14]. The kidneys handle most uric acid excretion, which is a metabolic byproduct of purine metabolism. Increased uric acid levels indicate disruptions in purine metabolism and renal function and are associated with gout, metabolic syndrome, and cardiovascular diseases.

## Materials and methods

Study setting

This cross-sectional study was conducted at the Central Research Laboratory, Jawaharlal Nehru Medical College, Datta Meghe Institute of Higher Education and Research, Sawangi Meghe, Wardha, India, with participants selected from Acharya Vinoba Bhave Rural Hospital. The institutional ethics committee approved the study protocol (No. DMIHER(DU)/IEC/2024/65).

Inclusion and exclusion criteria

Participants of either gender and aged between 18 and 65 years were eligible for the study. All participants were enrolled from January 2024 to April 2024 after obtaining the informed consent form. Both healthy individuals and patients attending the outpatient clinic who were willing to participate and had no history of cardiovascular disease (coronary heart disease, angina, arrhythmia, unstable angina) were screened and enrolled in the study.

Sample collection

Venous blood samples (10 mL) were collected from all participants after an overnight fasting period for biochemical analysis. Demographic data and other details were collected by using self-administered questionnaires.

Anthropometric measurements

Anthropometric measurements, including height in cm, using a stadiometer (PRESTIGE SM-P-W-210, India) and weight in kg using Healthgenie HD-221 Weight Machine, China, were measured, and body mass index (BMI) was calculated using a standard formula represented by kg/m^2^ unit.

BP evaluation

BP was measured using a sphygmomanometer (Omron HEM 7124 Fully Automatic Digital Blood Pressure Monitor, Japan). Participants were directed to rest for a minimum of five minutes while seated before the measurements were conducted. Systolic blood pressure (SBP) and diastolic blood pressure (DBP) were documented as the mean of two successive readings at two-minute intervals.

Biochemical analysis

Metabolic markers, including fasting blood glucose levels, total cholesterol levels, triglyceride levels, HDL cholesterol levels, GGT activity measured in international units per liter (IU/L), and uric acid levels, were analyzed using the MINI VIDAS system (bioMerieux SA, situated at 376, Chemin de l'Orme, 69280 Marcy-l'Étoile, France).

Statistical analysis

Statistical analysis was conducted using IBM SPSS Statistics for Windows, Version 17 (Released 2008; IBM Corp., Armonk, New York, United States). Descriptive statistics were computed for demographic and clinical characteristics, with continuous variables reported as means ± standard deviations (SD) and categorical variables as frequencies. Pearson correlation coefficients were calculated to evaluate the associations between cardiovascular health indicators (SBP, DBP) and metabolic markers (fasting blood sugar, lipid profile, BMI, GGT, and uric acid). A p-value greater than 0.05 (p < 0.05) is statistically significant.

## Results

SBP and DBP have a significant positive correlation (Pearson correlation coefficient = 0.919, p < 0.001). This suggests that DBP tends to increase in tandem with SBP. Table 1 displays noteworthy positive correlations between SBP and other cardiovascular risk factors, including low-density lipoprotein (LDL) cholesterol (0.727), total cholesterol (0.819), and triglycerides (0.818), all of which have p-values less than 0.001.

**Table 1:** Analysis of different metabolic parameters SBP: Systolic blood pressure; DBP: diastolic blood pressure; BP: blood pressure; BMI: body mass index; Fasting B. S.: fasting blood sugar; GGT: gamma-glutamyl transferase; HDL: high-density lipoprotein; "-": for blank space; "*": p-value more than 0.05 is considered as statistically nonsignificant (p > 0.05);   "**": p-value less than 0.05 is considered as statistically significant (p > 0.05)

		Age	SBP	DBP	Fasting B.S. mg/dl	Total cholesterol mg/dl	Triglycerides mg/dl	HDL mg/dl	Height cm	Weight kg	BMI Kg/m2	GGT IU/L	Uric acid mg/dl
Age	Pearson correlation	1	.310^*^	.328^*^	.176	.130	.180	-.065	-.312^*^	-.107	.235	.125	.193
	p-value	-	.028	.020	.220	.368	.210	.656	.027	.461	.101	.386	.179
SBP	Pearson correlation	-	1	.919^**^	.819^**^	.818^**^	.727^**^	-.544^**^	-.258	.278	.728^**^	.778^**^	.754^**^
	p-value	-	-	.000	.000	.000	.000	.000	.070	.051	.000	.000	.000
DBP	Pearson correlation	-	-	1	.729^**^	.759^**^	.673^**^	-.433^**^	-.297^*^	.200	.724^**^	.700^**^	.703^**^
	p-value	-	-	-	.000	.000	.000	.002	.036	.163	.000	.000	.000
Fasting B.S. mg/d	Pearson correlation	-	-	-	1	.800^**^	.754^**^	-.742^**^	-.086	.269	.463^**^	.908^**^	.709^**^
	p-value	-	-	-	-	.000	.000	.000	.552	.059	.001	.000	.000
Total cholesterol mg/dl	Pearson correlation	-	-	-	-	1	.733^**^	-.495^**^	-.062	.453^**^	.676^**^	.771^**^	.758^**^
	p-value	-	-	-	-	-	.000	.000	.666	.001	.000	.000	.000
Triglycerides mg/dl	Pearson correlation	-	-	-	-	-	1	-.623^**^	-.165	.284^*^	.595^**^	.740^**^	.720^**^
	p-value	-	-	-	-	-	-	.000	.252	.046	.000	.000	.000
HDL mg/dl	Pearson correlation	-	-	-	-	-	-	1	.020	-.175	-.240	-.732^**^	-.545^**^
	p-value	-	-	-	-	-	-	-	.889	.223	.093	.000	.000
Height cm	Pearson correlation	-	-	-	-	-	-	-	1	.614^**^	-.379^**^	-.133	-.114
	p-value	-	-	-	-	-	-	-	-	.000	.007	.356	.430
Weight kg	Pearson correlation	-	-	-	-	-	-	-	-	1	.412^**^	.279^*^	.421^**^
	p-value	-	-	-	-	-	-	-	-	-	.003	.049	.002
BMI kg/m2	Pearson correlation	-	-	-	-	-	-	-	-	-	1	.509^**^	.644^**^
	p-value	-	-	-	-	-	-	-	-	-	-	.000	.000
GGT IU/L	Pearson correlation	-	-	-	-	-	-	-	-	-	-	1	.764^**^
	p-value	-	-	-	-	-	-	-	-	-	-	-	.000
Uric acid mg/dl	Pearson correlation	-	-	-	-	-	-	-	-	-	-	-	1
	p-value	-	-	-	-	-	-	-	-	-	-	-	-
	N	-	-	-	-	-	-	-	-	-	-	-	-

This implies that poor lipid profiles are linked to elevated SBP, which increases the risk of cardiovascular illnesses. Similarly, DBP had significant positive correlations (p < 0.001) with LDL cholesterol (0.724), triglycerides (0.673), and total cholesterol (0.759). This supports the notion that negative lipid profiles and DBP are related to higher risk of cardiovascular disease.

Table 1 displays a strong positive correlation between fasting blood glucose and LDL cholesterol (0.908) and total cholesterol (0.800). The link between high blood glucose levels and adverse lipid profiles, which are important risk factors for cardiovascular diseases. Table 1 shows significant positive correlations between BMI and p-values < 0.001 for SBP (0.778), DBP (0.700), total cholesterol (0.676), triglycerides (0.595), and LDL cholesterol (0.644). This demonstrates the correlation between many cardiovascular risk factors and obesity, as measured by BMI. Table 1 indicates that GGT and uric acid have a significant positive correlation (0.764, p < 0.001), indicating a possible connection between uric acid metabolism and liver function. 

## Discussion

The present study showed a strong correlation between metabolic markers and cardiovascular health indicators, such as SBP and DBP. Raised BP was found to be a significant predictor of cardiovascular risk, which is in line with earlier research and emphasizes the significance of managing hypertension to prevent CVDs [15]. Atherosclerosis, endothelial dysfunction, and arterial stiffness are all facilitated by hypertension, which can eventually result in harmful cardiovascular outcomes like myocardial infarction and ischemic stroke [16]. Furthermore, our results highlighted the interaction between BP and metabolic variables, including lipid profile parameters and fasting blood glucose levels. One known risk factor for atherosclerotic CVD is dyslipidemia, which is defined as having decreased levels of HDL and elevated levels of triglycerides and total cholesterol [17]. The correlations between lipid profile parameters and BP support the theory that atherogenic dyslipidemia plays a role in the development of hypertension-induced cardiovascular damage [18]. Investigating the complex interactions between metabolic markers and cardiovascular health indicators is a crucial first step in improving our knowledge of cardiometabolic disorders and developing tailored strategies for risk assessment and treatment. Through the clarification of the intricate interactions between physiological, biochemical, and lifestyle factors, the objective of this research is to create focused interventions that will decrease the prevalence of metabolic disorders and cardiovascular diseases worldwide. The study data evaluate not only the lipid profile parameters but also BMI, an anthropometric indicator of adiposity, and specific metabolic enzymes like uric acid and GGT. Excessive accumulation of adipose tissue is a hallmark of obesity, which is closely associated with insulin resistance, dyslipidemia, and systemic inflammation, all of which increase the risk of cardiovascular disease [19]. The substantial relationships found between BP and BMI indicators emphasize the role that obesity plays in the etiology of hypertension and its associated cardiovascular consequences [20].

Furthermore, GGT, an enzyme involved in glutathione metabolism, has been linked to oxidative stress and liver dysfunction, with elevated levels associated with increased cardiovascular risk and mortality. The addition of uric acid and GGT as metabolic markers offers insights into their potential contributions to cardiometabolic health [21]. Although the exact mechanisms underlying these links remain unknown, uric acid, a metabolic byproduct of purine metabolism, has been linked to hypertension, endothelial dysfunction, and atherosclerosis [22]. The observed associations between these metabolic markers and cardiovascular health indicators indicate their potential use as supplementary risk stratification instruments in clinical settings. It is crucial to compare our results with those of earlier studies conducted in comparable environments to put them into context.

An increasing amount of research supports the multifactorial nature of cardiometabolic diseases and the significance of integrated approaches to risk assessment and management [23]. Previous studies have indicated correlations between conventional cardiovascular risk factors such as dyslipidemia and hypertension and metabolic disorders such as obesity and insulin resistance [24]. However, the present research focuses on risk factors associated with investigating new metabolic pathways and biomarkers related to cardiovascular and metabolic health. Numerous recent studies have investigated the connections between cardiovascular outcomes and metabolic enzymes like uric acid and GGT offering insights into possible mechanistic pathways [25] that have been implicated in oxidative stress, inflammation, and endothelial dysfunction, contributing to atherosclerosis and cardiovascular events [26]. Similarly, uric acid's association with hypertension, vascular dysfunction, and renal impairment underscores its potential as a target for treatment in cardiometabolic disorders [27].

The present study have many potential benefits including practical implications for managing, preventing, and assessing the risk of cardiometabolic disorders highlighted by the strong correlations between metabolic markers and cardiovascular health indicators. Anthropometric indices, BP readings, lipid profile parameters, metabolic enzymes, and other data from various domains can be integrated to provide thorough risk assessment and customized interventions [28]. Furthermore, our results demonstrate the potential value of new biomarkers for cardiovascular risk assessment, like uric acid and GGT. By adding these markers to current risk prediction models, it may be possible to increase the models’ discriminatory power and accuracy, which would allow for more accurate identification of people at high risk of developing cardiometabolic diseases [29]. Furthermore, targeted interventions that target modifiable risk factors such as obesity, dyslipidemia, and hypertension are crucial for both primary and secondary prevention of metabolic disorders and CVDs [30]. In addition, research on the molecular pathways underlying cardiometabolic disorders through experiments may reveal new therapeutic targets and approaches to enhance results [31]. Furthermore, methods for personalized or precision medicine that consider lifestyle, environmental, and genetic factors show promise for developing customized risk assessment and management plans [32].

Limitations

The cross-sectional design of the present study makes it impossible to establish causal links between the variables, and the associations that are found may be impacted by residual confounding, and further investigation is required to confirm our findings in terms of risk factor involved during the intervention time. Another limitation is the small sample size; the current study only includes 50 participants.

## Conclusions

In conclusion, our study revealed strong correlations between metabolic markers and cardiovascular health indicators, underscoring their role in assessing CVD risk. Notably, elevated SBP correlates significantly with adverse lipid profiles, emphasizing the importance of monitoring both SBP and DBP in cardiovascular health assessment. In addition, the positive associations between fasting blood glucose levels, BMI, and cardiovascular risk factors highlight the significance of glucose control and obesity management in reducing cardiovascular risk. Furthermore, the correlation between GGT and uric acid levels suggests potential connections to hepatic function, indicating complex physiological mechanisms. These findings advocate for targeted interventions and comprehensive evaluations to mitigate the burden of CVD and improve metabolic health. However, clarifying the underlying mechanisms that connect metabolic markers and cardiovascular health indicators should be included in future research.
